# Early Delivery of Misfolded PrP from ER to Lysosomes by Autophagy

**DOI:** 10.1155/2013/560421

**Published:** 2013-12-17

**Authors:** Constanza J. Cortes, Kefeng Qin, Eric M. Norstrom, William N. Green, Vytautas P. Bindokas, James A. Mastrianni

**Affiliations:** ^1^Departments of Neurology, MC2030, The University of Chicago Pritzker School of Medicine, 5841 S. Maryland Avenue, Chicago, IL 60637, USA; ^2^Departments of Neurobiology, The University of Chicago Pritzker School of Medicine, Chicago, IL 60637, USA

## Abstract

Prion diseases are linked to the accumulation of a misfolded isoform (PrP^Sc^) of prion protein (PrP). Evidence suggests that lysosomes are degradation endpoints and sites of the accumulation of PrP^Sc^. We questioned whether lysosomes participate in the early quality control of newly generated misfolded PrP. We found PrP carrying the disease-associated T182A mutation (Mut-PrP) was delivered to lysosomes in a Golgi-independent manner. Time-lapse live cell imaging revealed early formation and uptake of GFP-tagged Mut-PrP aggregates into LysoTracker labeled vesicles. Compared with Wt-PrP, Mut-PrP expression was associated with an elevation in several markers of the autophagy-lysosomal pathway, and it extensively colocalized with the autophagosome-specific marker, LC3B. In autophagy deficient (ATG5^−/−^) mouse embryonic fibroblasts, or in normal cells treated with the autophagy-inhibitor 3-MA, Mut-PrP colocalization with lysosomes was reduced to a similar extent. Additionally, 3-MA selectively impaired the degradation of insoluble Mut-PrP, resulting in an increase in protease-resistant PrP, whereas the induction of autophagy by rapamycin reduced it. These findings suggest that autophagy might function as a quality control mechanism to limit the accumulation of misfolded PrP that normally leads to the generation of PrP^Sc^.

## 1. Introduction

Prion diseases, such as Creutzfeldt-Jakob disease (CJD) of humans and bovine spongiform encephalopathy (BSE) of cattle, are transmissible neurodegenerative disorders linked to the accumulation of a misfolded isoform (PrP^Sc^) of the host-encoded glycophosphatidylinositol (GPI)-linked prion protein (PrP^C^) [1]. As a membrane protein, PrP^C^ follows the secretory pathway to its destination on the outer leaflet of the plasma membrane where it ultimately follows the endocytic pathway for degradation in lysosomes. Mutations of the PrP gene linked to familial prion disease promote the misfolding of PrP that may delay its exit from the endoplasmic reticulum, leading to impaired delivery to the plasma membrane and an alternative pathway for degradation.

Autophagy is an evolutionarily conserved lysosomal degradation pathway usually activated under low nutrient conditions which acts to sequester and deliver cytoplasmic material, including organelles, toxic metabolites, or intracellular pathogens, to the lysosome for degradation and/or recycling [2]. This process is highly regulated by a series of autophagy-related gene products or Atg proteins [3, 4]. Key proteins include Atg6 and its mammalian homolog Beclin-1, which participate in the formation of the double layered isolation vacuole [5], Atg8 and its mammalian homolog, the cytosolic microtubule associated protein 1 light chain 3 (MAP-LC3) that is incorporated into autophagosome membranes [6], and Atg12 and Atg5, which are required for autophagosomal membrane nucleation and are targeted to autophagosomes via a ubiquitin-like conjugation system [7]. In the recent years, autophagy has been shown to function in the elimination of several neurodegenerative-linked proteins [8–10], including PrP [11–13].

We recently found that chronic administration of the autophagy-inducing agent rapamycin to transgenic Tg(PrP-A116V) mice that model genetic prion disease, reduced the total load of misfolded PrP, prevented PrP amyloid plaque deposition in their brains, and significantly delayed disease onset [13]. These results support autophagy as a mechanism to limit the production of misfolded PrP; however, the cellular pathway by which misfolded PrP is eliminated has not been defined. To begin to address this question, we studied the possible role of autophagy in the cellular trafficking of a familial CJD-associated PrP mutant (T183A) [14, 15], well known to undergo intracellular aggregation and accumulation [16–20]. Our findings suggest that autophagy functions as an early quality control mechanism to limit the *de novo* generation of misfolded pathogenic PrP.

## 2. Materials and Methods 

### 2.1. Plasmids/Cell Culture

The T182A mutation was introduced into a mouse sequence wild type (Wt) PrP containing pSP72 plasmid, using Quick Change (Stratagene) and the following PCR primers: GACTGCGTCAATATCGCCATCAAGCAGCACACG (T182A sense), CGTGTGCTGCTTGATGGCGATATTGACGCAGTC (T182A anti). An additional set of Wt and T182A mutant PrPs was engineered with the human/hamster monoclonal antibody (mAb) 3F4 epitope, to allow selective detection of recombinant mouse PrP in mouse neuroblastoma N2a cells that express moderate levels of endogenous PrP. The PrP-GFP construct, a gift of David Harris (Boston University, Boston, MA), was also used to generate Wt and T182A PrPs lacking the 3F4 epitope. All constructs to be expressed in mammalian cells were ligated into the pCB6^+^ vector under the control of the CMV promoter, using standard molecular biological protocols. Cell lines were purchased from ATCC and grown in recommended media at 37°C and 5% CO_2_. For transfections, Lipofectamine 2000 (Invitrogen), following manufacturer's protocol, was used. For stable transfections, cells were selected and maintained in 200 *μ*g/mL G418 (Sigma) added to the media. Reagents used were bafilomycin (BafA1) 0.05–0.1 *μ*M (Sigma), brefeldin A (BFA) 5–10 *μ*g/mL (Sigma), 3-methyladenine (3-MA) 2–10 mM (Sigma), rapamycin 1–20 *μ*M (Sigma), leupeptin 100 *μ*M (Sigma), and E-64 20 *μ*M (Sigma). Endo H (New England Biolabs) digestions were performed according to the manufacturer's instructions. To ensure the results were not specific to a single cell type, we employed three cell types for these studies: mouse neuroblastoma N2a, HeLa, and COS-7 cells. Of this group, N2a cells best approximate neurons, making them most useful for the fractionation studies and other functional studies such as assessing autophagy upregulation. However, because of their small cytoplasm, they were not ideal for imaging studies. For these, HeLa cells, with their larger cytoplasm, and which have been used extensively in autophagy-related studies elsewhere, were primarily used. COS-7 cells were also used in the extended time lapse live imaging studies, as these are large cells with a flat architecture, and they provide the best option for time-lapse imaging. Because they lack endogenous PrP expression, they were also employed in many of the biochemical assays.

### 2.2. Antibodies

The D13 human F(ab) anti-mouse PrP antibody (InPro) was used to detect recombinant mouse PrP expressed in HeLa cells, and the 3F4 mAb (gift of Richard Kascsak, Staten Island, NY) was used in N2a cells expressing recombinant PrP engineered with the 3F4 epitope tag (residues 109 and 112 as Met). Cy5 conjugated anti-human or anti-mouse antibody (Jackson ImmunoResearch) was used as the secondary antibody for immunofluorescence. Primary antibodies against giantin and secondary antibodies AlexaFlour 488 anti-mouse, anti-rat, and anti-rabbit were from Molecular Probes. For Western blots, HRP-conjugated anti-human (D13) or anti-mouse (3F4) IgG (Pierce) was used. Primary antibodies LAMP-1, BECN-1, and tubulin, in addition to secondary anti-mouse IgM and anti-mouse IgG secondary antibodies, were purchased from Santa Cruz Biotechnology. LC3B polyclonal rabbit primary antibody and phosphorylated mTOR and Atg12 polyclonal antibodies were from Cell Signaling.

### 2.3. Immunolocalization

In most cases, cells were grown on glass coverslips, fixed with ice cold methanol for 5 minutes, blocked in 2% BSA in PBS, probed (overnight at 4°C in a wet chamber) with appropriate primary antibody dilutions (1 : 50–1 : 200) in PBS, and incubated for 1 hr at 23°C with secondary antibody (1 : 200) in PBS, and appropriate washing throughout. Coverslips were mounted with antifade medium (Vectashield) and stored in the dark (4°C). Specimens were imaged on an Olympus DSU spinning disk confocal microscope system. For double-immunostaining studies, sequential scanning as well as secondary antibodies with distinct emission spectra (Cy5 and Alexa488) was used to eliminate fluorescence crosstalk between color channels. For LC3 staining and colocalization with GFP-tagged PrP, cells were fixed in 4% paraformaldehyde in PBS at room temperature for 15 min, washed with PBS, permeabilized in 0.1% Triton X-100 for 5 minutes, washed in PBS, blocked in 2% BSA for 1 h, and then incubated at 4°C overnight with rabbit anti-LC3 antibody (Cell Signaling) at 1 : 200. Following a wash, cells were incubated with secondary antibodies and DyLight 649-conjugated AffiniPure goat anti-rabbit IgG (Jackson ImmunoResearch, Lab, Inc.) (1 : 200) at room temperature for 3 h, and then washed with PBS. For staining nuclei, cells were incubated with 10 *μ*g/mL DAPI (Sigma-Aldrich) for 1 min. Cells were analyzed using NIH ImageJ software. Li's method for the estimation of colocalization was determined using an ImageJ software JACOP plugin. Li's intensity correlation quotient (ICQ) provides an overall index of whether the staining intensities of two channels are associated in a random, dependent, or segregated manner. It ranges from 0.5 (completely dependent correlation) to −0.5 (completely segregated staining) [21].

### 2.4. Live Cell Imaging

For short term visualization, LysoTracker DND-99 (Molecular Probes), at a final concentration of 50 nM, was added to media 30 minutes prior to imaging. For time-lapse imaging, the concentration of LysoTracker was reduced to 15–30 nM to prevent lysosomal dye overload. Confocal photomicroscopy was performed on an Olympus IX81 DSU spinning disk confocal microscope equipped with a 100x (NA 1.45) oil objective, a 14-bit chilled EM-CCD camera (Hamamatsu C9100-12), Ludl filter changers/shutters, and a stage microincubator with 5% CO_2_ (Harvard Apparatus). Z stacks were collected every 1 micrometer over the cell volume once, for steady state imaging, or every 10 min for 5 to 16 h, to capture protein expression, using Slidebook software. Multiple fluorophore acquisitions used sequential capture to avoid crosstalk.

### 2.5. Electron Microscopy

Cells were postfixed with 4% paraformaldehyde (PFA) plus 1.25% glutaraldehyde for 60 min, rinsed with 0.1 M sodium cacodylate buffer, and fixed with 1% OsO_4_ in sodium cacodylate (pH 7.4, 1 h at 4°C). Osmicated cells were rinsed with maleate buffer and then en bloc stained with 1% uranyl acetate in maleate buffer, pH 6.0, for 1 h. Cells were dehydrated in graded ethanol solutions and then embedded in Spurr's resin. Cells were thin sectioned, stained with lead citrate and uranyl acetate, and examined using a FEI Tecnai F30 electron microscope.

### 2.6. Subcellular Fractionation

N2a cells from two 100 mm dishes were homogenized using a ball-bearing homogenizer with a 12 *μ*m clearance in 0.25 M sucrose, 10 mM Tris-HCl (pH 7.4), 1 mM MgAc_2_, and a protease inhibitor cocktail (Roche) at a final concentration of 1 vol of cell pellet per 5 volumes of homogenizing medium. Postnuclear supernatants were fractionated by top loading on a step sucrose gradient comprised of 1 mL of 2 M sucrose, 4 mL of 1.3 M sucrose, 3.5 mL of 1.16 M sucrose, and 2.0 mL of 0.8 M sucrose. All solutions contained 10 mM Tris-HCl, pH 7.4, and 1 mM MgAc_2_. The gradients were centrifuged for 2.5 h at 100,000 ×g in a Beckman SW41Ti rotor. Twelve 1 mL fractions were collected from the top of each gradient and assayed by Western blot.

### 2.7. Western Blotting

As previously described [22], confluent cells were lysed in ice cold lysis buffer (mM: 20 Tris-HCl, pH 8.0, 150 NaCl, 1 EDTA; 0.5% Triton X-100, 0.5% Na-DOC), protein concentration determined with BCA assay (Pierce), and 30–50 *μ*g total protein subjected to SDS PAGE. Proteins were transferred to PVDF membranes (BioRad), probed with primary antibodies (described in text) overnight, washed and incubated with HRP-conjugated secondary antibodies (1 : 5000 dilution; 60 min), and developed with West Pico ECL (Thermo Scientific, Rockford, EL) chemiluminescence reagent for 5 min. Blots were exposed using a BioRad XRS Image Documentation system, and densitometry was assessed by ImageJ.

### 2.8. Solubility Assay

Cells were lysed in lysis buffer, cleared at 1500 ×g for 1 minute, then centrifuged at either 16,000 ×g (Figure 4) or 100,000 ×g (Figure 5) for 1 hour at 4°C. The supernatant was separated and the pellet was washed, centrifuged, and resuspended in the original starting volume of lysis buffer. Equal volumes of reconstituted pellet and supernatant were submitted for Western analysis. Fractions of soluble and insoluble PrP were estimated as the densitometric fraction of the sum of the supernatant and pellet fraction signals, as described in the text.

### 2.9. PIPLC Release Assay

Confluent cells were transiently transfected with either Wt- or Mut-PrP and expressed overnight in 150 mm plates (~2.6 × 10^7^ cells). At the begining of the experiment the cells were washed 5 times with Opti Pro SFM (serum free media) and then incubated with 3 mL of the same media with or without PIPLC at 1 U/mL (Sigma, Cat number p-8804) at 4°C for 90 min. The release medium was collected and cleared of detached cells by centrifugation at 900 rpm for 3 min. Total protein was precipitated from the media by the addition of 4 volume of cold methanol and centrifugation at 12,000 ×g for 10 min at 23°C. The pellet was dried and resuspended in 30 mL of 1X SDS loading buffer, all of which was subjected to SDS-PAGE and immunoblotting. The cells on the plates were rinsed twice with PBS and harvested by the addition of lysis buffer for Western analysis.

## 3. Results

### 3.1. PrP-T182A Is Aberrantly Trafficked

**Figure 1:**
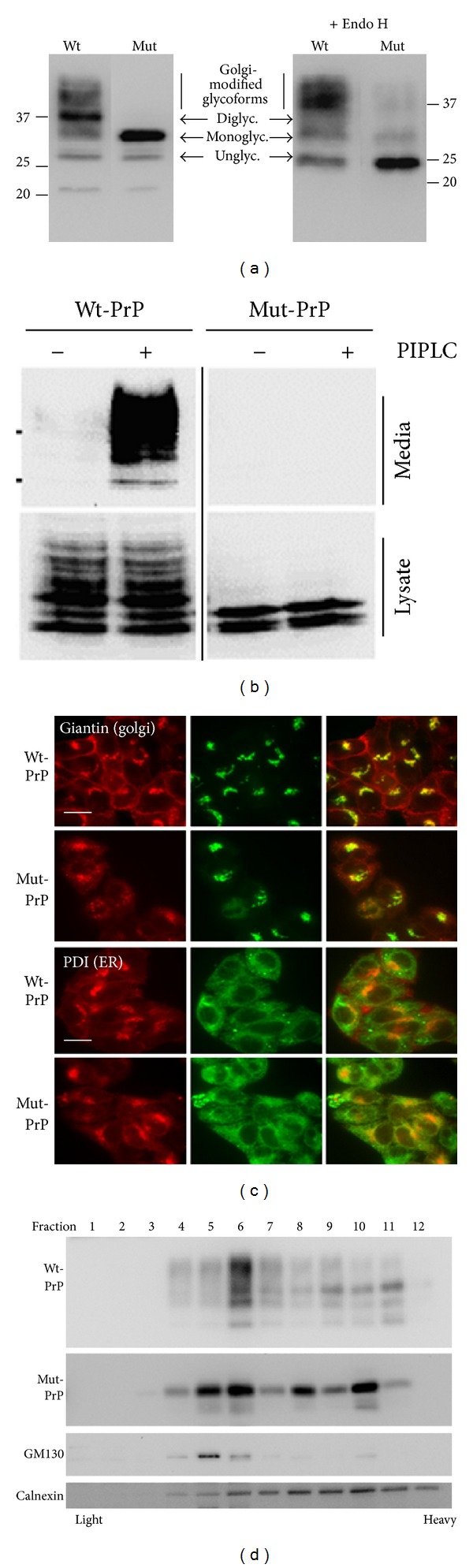
Aberrant trafficking of PrP-T182A. (a) Western blot of whole cell lysates prepared from N2a cells transiently expressing either Wt- or Mut-PrP carrying the 3F4 epitope (i.e., PrP(3F4)) for 16 h, before and after digestion with Endoglycosidase H (Endo H), followed by probing with 3F4 mAb. Wt-PrP has two N-linked glycosylation sites at positions 180 and 196, producing un-, mono-, and diglycosylated PrP. The T182A mutation disrupts the N-X-T glycosylation consensus site, resulting in loss of diglycosylated PrP. The lower molecular weight fractions of Wt-PrP and the core-glycosylated fraction of Mut-PrP are sensitive to Endo H, whereas higher molecular weight, Golgi-modified glycoforms, is resistant. (b) Western blot of Wt- and Mut-PrP recovered from the media (top) or lysates (bottom) of COS-7 cells transiently expressing PrPs for 16 h and then incubated for 90 min at 4°C in OptiPro media in the absence (−) or presence of PIPLC (+). Wt-PrP was detected in the media of PIPLC treated cells, whereas Mut-PrP was not (top panels), although the expression of each was evident in cell lysate preps (bottom panels), as revealed by D13 F (ab) anti-mouse-PrP antibody. (c) Immunofluorescence confocal microscopy of N2a cells expressing either Wt- or Mut-PrP(3F4) (red), costained for giantin or PDI (green). In contrast to Wt-PrP, Mut-PrP is consistently absent from the plasma membrane although it does label the ER and Golgi, indicating limited trafficking to at least cis-Golgi. Scale bars = 12 *μ*m. (d) Sucrose gradient subcellular fractionation of N2a cells stably expressing Wt- or Mut-PrP(3F4) shows that Mut-PrP is distributed primarily in heavier fractions that correspond with the ER marker calnexin, compared with Wt-PrP that cofractionates primarily with the Golgi marker GM130. PrP is detected with 3F4 mAb.

The alanine substitution of threonine at residue 182 of mouse PrP (183 in human PrP) disrupts the N-X-T consensus sequence for the first of two N-linked glycosylation sites, which eliminates the diglycosylated fraction of PrP and results in its propensity to aggregate and undergo aberrant trafficking [16]. Several investigators have shown that PrP-T182A does not traffic to the plasma membrane [19, 20, 23, 24], which was confirmed in our hands, using three separate cell lines, including HeLa, N2a, and COS-7 cells. Representative examples of studies replicated in at least two cell types are presented. First, PrP-T182A (hereafter referred to as Mut-PrP) transiently expressed for 16 h produced only non- and monoglycosylated PrP that were sensitive to Endoglycosidase H (N2a data, using 3F4 tagged PrPs is presented), supporting a lack of complex glycosylation and limited trafficking to cis-Golgi (Figure 1(a)); second, phosphatidylinositol-phospholipase C (PIPLC) treatment, which cleaves PrP from its GPI anchor, effectively released surface-bound Wt-PrP, but not Mut-PrP, into the culture media (COS-7 cell data, using nontagged PrPs, presented) (Figure 1(b)); and third, immunofluorescence confocal microscopy showed Wt-PrP to be consistently present on the plasma membrane, whereas Mut-PrP was consistently absent (N2a data presented). However, both Wt-PrP and Mut-PrP colocalized with ER (protein disulfide isomerase, PDI) and Golgi (giantin) markers although we questioned whether Mut-PrP colocalized more completely with ER and less completely with Golgi than did Wt-PrP (Figure 1(c)). To assess a potential difference in steady state cellular distribution, we performed sucrose gradient cell fractionation on N2a cells stably expressing Wt- or Mut-PrP (Figure 1(d)). The vast majority of Wt-PrP was present within fraction 6, consistent with Golgi (GM130), whereas Mut-PrP was found in heavy and light fractions although it was concentrated primarily within the heavier fractions, especially fractions 8 and 10 that cofractionated with the ER marker calnexin (Figure 1(d)). The presence of Mut-PrP in lighter fractions is not surprising, based on its cycling within the cis-Golgi. The apparent discontinuous enrichment in fractions 8 and 10 suggests they might be compartmentalized as aggregates in variably sized vesicles.

**Figure 2:**
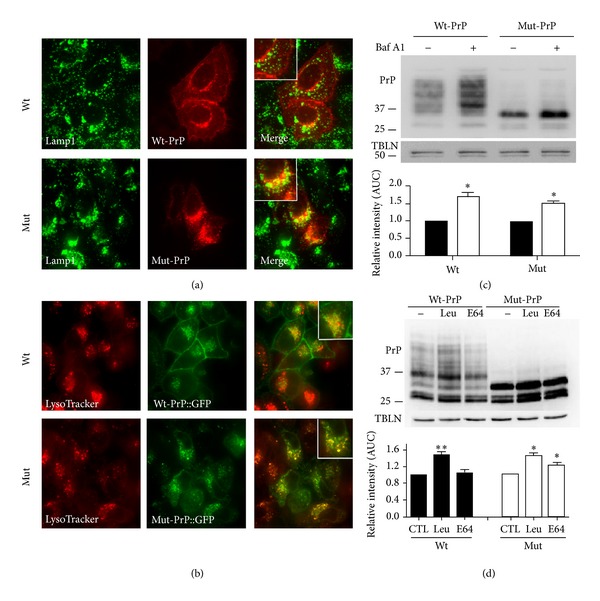
Mut-PrP in lysosomes. (a) Indirect immunofluorescence confocal microscopy of fixed HeLa cells after 16 h expression of Wt- or Mut-PrP, detected with anti-mouse-PrP D13 F(ab) antibody. Red (PrP) and green (LAMP-1) channels are displayed separately to the left of merged images. Yellow indicates colocalization. (b) Live cell fluorescence microscopy of HeLa cells transiently expressing Wt-PrP::GFP or Mut-PrP::GFP in the presence of 50 nM LysoTracker Red shows comparable results with indirect immunoflourescence. (c) Western blot of total lysates of COS-7 cells transiently expressing Wt- or Mut-PrP after 16 h incubation with vehicle (−) or 0.1 *μ*M Bafilomycin A1 (Baf A1) added to the media. PrP is detected with the D13 antibody. Graphic representation of densitometric signal of Western blot from 3 experiments is displayed below. (d) as in (c), but following incubation in the absence (−) or presence of 100 *μ*M Leupeptin (Leu) or 20 *μ*M E64, probed with D13. *α*-tubulin (TBLN) as a loading control in (c) and (d). Molecular weight markers (kDa) on the left. Densitometric quantification of blots in (c) and (d) is shown in graphs as relative intensity compared to untreated condition. *P < 0.05, **P < 0.005 (Student's paired *t*-test, *n* = 3 experiments each).

To establish whether Mut-PrP is delivered to lysosomes despite the lack of plasma membrane localization, we performed indirect immunofluorescence confocal microscopy using HeLa cells following 16 h of transient expression of Wt- or Mut-PrP. HeLa cells provide an advantage to N2a cells, as they have a larger cytosol to facilitate the imaging of lysosomes and their colocalization with PrP. Lysosome-associated membrane protein-1 (LAMP-1) was found to colocalize with both Wt- and Mut-PrP, supporting the delivery of Mut-PrP to lysosomes (Figure 2(a)). To more conveniently assess its presence in lysosomes, we performed live imaging in HeLa cells expressing a GFP-tagged PrP (PrP::GFP) in the presence of LysoTracker Red, a fluorescent marker that preferentially accumulates within acidic vesicles that primarily include lysosomes (Figure 2(b)). The insertion of GFP between the last residue of mature PrP (position 230) and the GPI anchor does not alter its trafficking and permits live cell imaging [25]. To estimate the degree of the colocalization of PrP with LysoTracker, the raw images from red and green channels were applied to Li's intensity correlation quotient (ICQ), using an ImageJ (NIH) plugin. The ICQ ranges from −0.50 to +0.50, the former representing a completely random association and the latter representing a highly dependent association. We found that Wt-PrP::GFP intensely labeled the plasmalemma and colocalized sparsely with intracellular LysoTracker (ICQ of 0.19 ± 0.011) (Figure 2(b), top row), whereas Mut-PrP::GFP was undetectable on the plasmalemma and accumulated intracellularly as puncta that colocalized extensively with LysoTracker (ICQ of 0.48 ± 0.02, *P* < 0.001, student's *t*-test, *n* = 15 cells) (Figure 2(b), bottom row). Because the pattern of colocalization with LysoTracker was similar to that observed with LAMP-1, we considered it to be a surrogate marker for lysosomes although we recognize that other acidic vesicles may be labeled.

To establish whether Mut-PrP undergoes active degradation in lysosomes, we assessed the effect of lysosome inhibitors on the steady state level of Mut-PrP. Here, we used COS-7 cells, which conveniently lack endogenous PrP expression allowing the expression of non-3F4-tagged mouse PrPs. Cells were transiently transfected and allowed to express Wt- or Mut-PrP for 16 h prior to incubation for 6 hours with the vacuolar-type ATPase inhibitor Bafilomycin A1 (Baf A1) (Figure 2(c)), E-64, or leupeptin, inhibitors of resident lysosomal enzymes cathepsins B and L (Figure 2(d)). In addition to the expected increase in Wt-PrP, a significant increase in the steady state level of Mut-PrP was seen with each inhibitor, suggesting that it undergoes some degree of degradation in lysosomes.

### 3.2. Mut-PrP Follows a Direct Intracellular Route to Lysosomes

**Figure 3:**
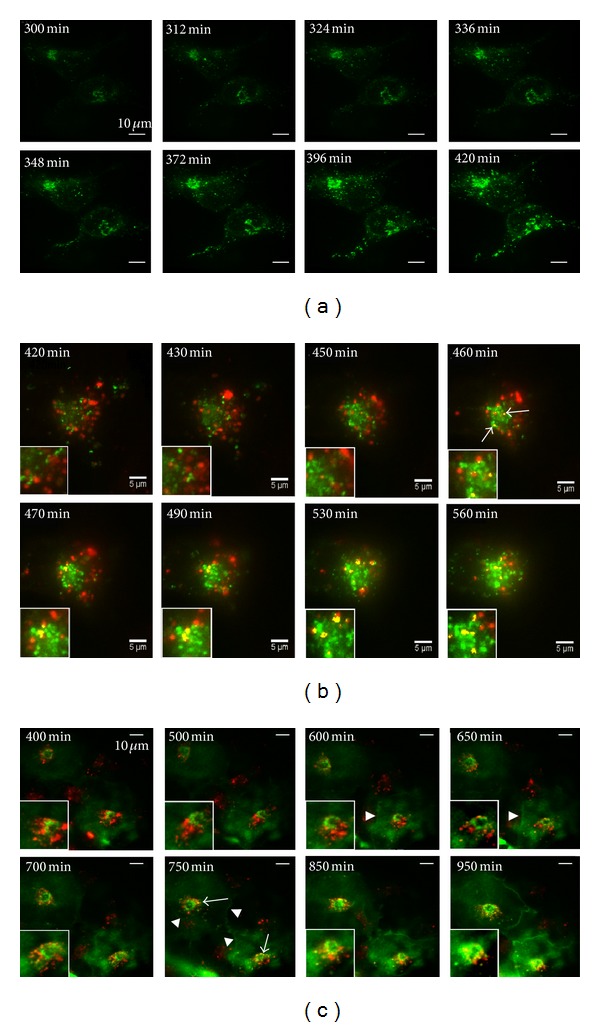
Time-lapse photomicroscopy of PrP::GFP trafficking to lysosomes. (a) At *T* = 0 COS-7 cells were transfected with Mut-PrP::GFP over 3 h, the media were replaced, and imaging started. (a) displays frames of live video collected from *T* = 300 min (5 h) to 420 min. Images were extracted as individual frames from supplement Video S1, using ImageJ. (b) Mut-PrP::GFP, as in (a), but in the presence of 30 nM LysoTracker Red. Frames were collected between *T* = 420 (7 h) and 560 min. Arrows indicate the first appearance of yellow puncta, signifying Mut-PrP delivery to LysoTracker Red positive vesicles. Images were extracted from supplement Video S2. Magnified insets, approximately 2x original image. (c) Wt-PrP::GFP in the presence of LysoTracker Red imaged as in (b) for Mut-PrP, but with a time window of 400 to 950 min, to capture PrP entry into lysosomes. Wt-PrP::GFP is detected on the plasmalemma (arrowheads) prior to its colocalization with LysoTracker Red positive vesicles (arrows), at a much delayed time point than Mut-PrP (750 versus 460 min). Images extracted from supplement Video S3. Magnified insets 4x original image, to compare with (b).

The absence of Mut-PrP on the plasmalemma and its colocalization with LAMP-1/LysoTracker positive vesicles support an intracellular route to lysosomes rather than the canonical endocytic pathway from plasmalemma to lysosomes. In support of this, we carried out time-lapse confocal microscopy of GFP-tagged PrP to track its course. COS-7 cells, based on their large size and flat architecture, features that facilitate time lapse imaging, were used for these studies. Cells were transiently transfected for 3 h, at which time the transfection media was replaced with fresh media and time-lapse imaging started. Mut- and Wt-PrP expression was detectable by 5 h (300 min) in some cells, but between 6 and 8 h in the majority of cells (90% of 120 cells surveyed). Thus, in some experiments, time-lapse imaging was delayed until 5 to 7 h, for efficiency. The general pattern of the expression of Mut-PrP was initially studied. As with other cell types, no plasma membrane localization could be appreciated in COS-7 cells. Surprisingly, at the earliest appearance of signal, Mut-PrP formed puncta that were distributed throughout the cell. With observation up to 12 h, the puncta grew in number, intensity, and size (Figure 3(a) and supplement Video S1 in Supplementary Materials available online at http://dx.doi.org/10.1155/2013/560421).

To visualize the intracellular delivery of Mut-PrP to acidic compartments, a similar paradigm as above was followed, but with 30 nM LysoTracker Red added to the media 30 min prior to imaging at ~7 h (Figure 3(b) and supplement Video S2). Within 8 h (480 min) following transfection, yellow puncta signifying the colocalization of Mut-PrP with LysoTracker Red positive vesicles were detected (Figure 3(b), arrows). Continued imaging revealed an increase in the number and size of yellow puncta over time, suggesting ongoing delivery and accumulation of Mut-PrP within acidic vesicles. In contrast to Mut-PrP, Wt-PrP appeared primarily as a diffuse signal although a small number of puncta that colocalized with LysoTracker Red positive vesicles appeared at a much later time point at ~750 min after transfection. Importantly, these puncta were observed after Wt-PrP was visualized on the plasmalemma at ~600–650 min (Figure 3(c) and supplement Video S3). Thus, these findings support the concept that Wt-PrP follows an indirect pathway that leads first to the plasma membrane and then lysosomes via the canonical endocytic pathway, whereas Mut-PrP is delivered to lysosomes via a faster, more direct intracellular route that does not involve the plasmalemma.

### 3.3. The Golgi Is Not Essential for This Pathway

**Figure 4:**
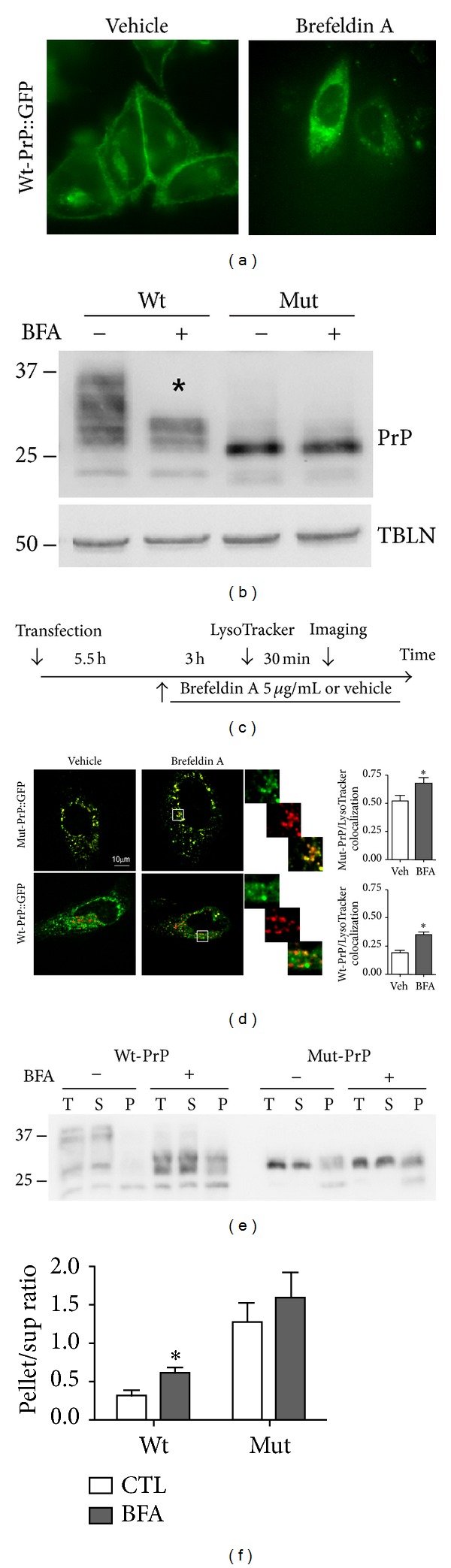
An intact Golgi is not essential for the delivery of Mut-PrP to LysoTracker positive vesicles. (a) Golgi disruption by Brefeldin A (BFA) (5 *μ*g/mL for 3 h) was confirmed by the absence of Wt-PrP signal on the plasmalemma of HeLa cells expressing Wt-PrP::GFP, compared with vehicle-treated cells sampled at the same time point. (b) Functional disruption of Golgi by BFA was biochemically confirmed by the absence of complex glycans (*) on Wt-PrP, following Western blot of total cell lysates, compared with vehicle-treated (−) cells. Mut-PrP does not acquire complex sugars and as such is unaffected by BFA. PrP was detected with D13 antibody. (c) Experimental paradigm; times are relative to the completion of the transfection protocol. BFA (5 *μ*g) was added at 5.5 hours, at the earliest detection of PrP signal, LysoTracker Red (50 nM) was added 30 minutes prior to the start of imaging at 9 hours, and images were collected by 10 hours. (d) Immunofluorescence of live cells expressing GFP-tagged Mut-PrP after treatment with vehicle or BFA. Green (PrP::GFP) and red (LysoTracker) channels are displayed as merged. Separate channels for the boxed areas of BFA treated cells are magnified and displayed to the right of the merged images. Correlation coefficient analysis revealed a statistically significant increase in colocalization of PrP and LysoTracker after BFA treatment for both Wt-PrP and Mut-PrP. Wt-PrP veh = 0.1891 ± 0.0194, BFA = 0.3505 ± 0.01997 (*n* = 28 cells, *P* < 0.001); Mut-PrP veh = 0.5112 ± 0.05329, BFA = 0.6710 ± 0.05438 (*n* = 23 cells, *P* < 0.05). (e) BFA treatment increases the insoluble fraction of PrP. N2a cells stably expressing 3F4-tagged Wt- or Mut PrP were treated with BFA (5 ug/mL for 3 h) or vehicle, and the insoluble fraction of PrP was separated by centrifugation at 16,000 ×g, and equal volumes of the total (T), supernatant (S), and pellet (P) fractions were loaded and subjected to Western blot using the 3F4 mAb. (f) Densitometric quantification of (e), using Image One (BioRad) software. The fraction of insoluble Wt-PrP was significantly increased after BFA treatment (*P* < 0.05,  *n* = 4), while the increase in insoluble Mut-PrP did not reach significance (*n* = 4).

It is well established that lysosomal targeting of ER-synthetized proteins occurs from the trans-Golgi network (TGN) via a mannose-6-phosphate receptor-mediated pathway [26]. However, based on its sensitivity to Endo H, PrP carrying the T182A mutation does not traffic beyond the mid-Golgi stack, making that route unlikely. This is supported by our findings and prior work from others that show this PrP mutant is retained in the ER [27], suggesting that it may not pass ER quality control. However, to determine whether the Golgi participates in this pathway, we assessed whether chemical disruption of Golgi by Brefeldin A (BFA) impairs the colocalization of Mut-PrP with LysoTracker labeled vesicles. To first confirm effective disruption of Golgi, we treated HeLa cells, transiently expressing either Wt-PrP::GFP or untagged Wt-PrP, with 5 *μ*g/mL of BFA for 3 hours, and assessed the effect on the trafficking and glycosylation of PrP. Functional disruption of Golgi was noted by, (1) the absence of Wt-PrP::GFP labeling on the plasma membrane (Figure 4(a)), (2) the presence of intracellular reticular staining pattern, consistent with ER retention and, (3) the absence of high molecular weight glycoforms normally added in the Golgi (Figure 4(b), asterisk).

We then assessed the effect of BFA on PrP colocalization with LysoTracker Red. HeLa cells were transfected with Wt- or Mut-PrP::GFP and allowed to express for 5.5 h (the earliest time point at which PrP signal was detected in our time lapse studies), at which time BFA or vehicle was added for 3 h. LysoTracker Red was added for 30 min, and cells were visualized by confocal fluorescence live cell microscopy at ~9 h after transfection (Figure 4(c)). In cells treated with BFA, Mut-PrP colocalization with LysoTracker not only persisted, but also increased slightly (*P* < 0.05) relative to cells with intact Golgi (Figure 4(d)). Interestingly, Golgi disruption also produced a small, but statistically significant, increase in Wt-PrP colocalization with LysoTracker (Figure 4(d)). We considered that, in the absence of functionally intact Golgi, Wt-PrP might be retained in the ER and, because of the general propensity of PrP to misfold, it too may aggregate within the ER lumen and follow the same pathway to lysosomes as Mut-PrP. In support of this, we found the detergent insoluble fraction of Wt-PrP was significantly increased in BFA-treated cells (Figures 4(e) and 4(f)). Interestingly, BFA did not significantly increase the already elevated fraction of insoluble Mut-PrP (Figure 4(f)). This provides further support that Mut-PrP undergoes limited trafficking to the Golgi and has a high propensity to aggregate in the ER independent of the Golgi. Overall, these findings suggest that (1) an intact Golgi is not required for the intracellular delivery of Mut-PrP to lysosomes; (2) the ER is the primary site of origin of Mut-PrP that accumulates in lysosomes; and (3) aggregation of PrP, induced by its retention within the ER, might trigger its intracellular transport to lysosomes.

### 3.4. Mut-PrP Expression Activates Autophagy

**Figure 5:**
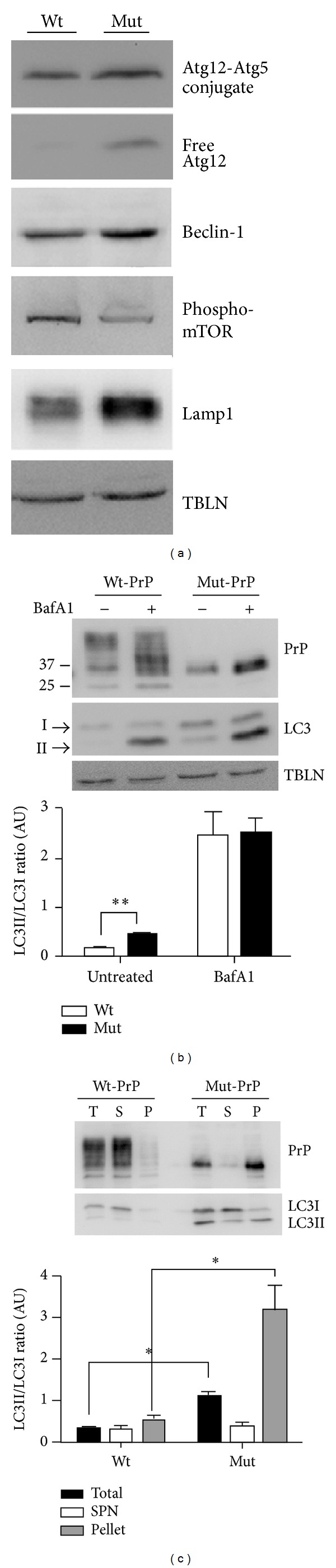
Mut-PrP expression activates autophagy. (a) Western blot of total lysates of N2a cells stably expressing Wt- or Mut-PrP engineered with the 3F4 epitope and probed for Atg12 (free and conjugated with Atg5), Beclin-1, phosphorylated mTOR, and LAMP-1. Loading was normalized for total protein. Tubulin (TBLN) is presented as a loading control. (b) Western blots of total lysates prepared from cells in (a) that were treated for 6 h with vehicle or 50 nM Bafilomycin A1 to limit LC3 degradation and probed for PrP with 3F4 mAb, LC3, and tubulin. PrP was detected by 3F4 mAb. The LC3II/LC3I ratio was measured by densitometry of each fraction, using Image One (BioRad) software from ECL developed Western blots, and displayed in the graph below (**P = 0.002, student's *t*-test, *n* = 3 experiments). (c) Representative blots of total (T), supernatant (S), and pellet (P) fractions of cells in (a), following lysis and separation by centrifugation at 100,000 ×g for 1 h, were probed for PrP and LC3. LC3II/LC3I ratios for each fraction are presented in the graph below (*P < 0.05, student's *t*-test,  *n* = 3).

Because autophagic vesicles have been reported by some to originate from ER membranes [28, 29] and Mut-PrP is retained within the ER, we considered autophagy as a possible mechanism for intracellular delivery of Mut-PrP to lysosomes. To initially screen for autophagy activation, we compared our stable N2a cell lines stably expressing Wt- or Mut-PrP, with respect to the steady state levels of several markers of the autophagy-lysosome pathway, including Beclin-1, an autophagy initiator and the mammalian homologue of Atg6, free Atg12 and Atg12 bound to Atg5, which are targeted to autophagosomes, and LAMP-1. Compared with Wt-PrP expressing cells, the level of each of these markers was greater in cells expressing Mut-PrP. In addition, the phosphorylated (activated) mammalian target of rapamycin (mTOR), which is normally suppressed during autophagy, was reduced in Mut-PrP cells relative to Wt-PrP expressors (Figure 5(a)).

Microtubule-associated protein 1 light chain 3 (MAP1-LC3), the homolog of Atg8 in yeast, referred to as LC3 in mammals, is specifically incorporated into autophagosomes [6, 30]. During autophagy LC3I, a 16 kDa protein is cleaved, lipidated, and incorporated into autophagosome membranes as LC3II, a 14 kDa protein. Thus, an increase in the LC3II/LC3I ratio has been used as a marker for autophagy induction [31]. We found this ratio to be significantly higher in cells expressing Mut-PrP compared to those expressing Wt-PrP (*P* = 0.002) (Figure 5(b)). An increase in LC3II might also result from impaired autophagic flux, resulting either from impaired fusion of the autophagosome with the lysosome or impaired degradation of LC3II within the lysosome. To indirectly test for this, we treated some cells with the vacuolar-type H(+)ATPase inhibitor Bafilomycin A1, which alkalinizes lysosomal compartments leading to impaired degradation of LC3II and/or reduced fusion of autophagosomes with lysosomes [32, 33]. Cells were incubated with 50 nM Bafilomycin A1 added to media for 6 h, prior to lysis and Western blotting. This resulted in a marked increase in the level of LC3II in both cell lines, suggesting that autophagic flux is not obviously impaired in Mut-PrP expressors (Figure 5(b), graph).

To determine if the increased LC3II was associated with the misfolded/aggregated fraction of Mut-PrP, we separated detergent soluble and insoluble fractions of these cells, by centrifugation at 100,000 ×g, and compared the LC3II/LC3I ratios within each fraction (Figure 5(c)). As expected, Mut-PrP was primarily confined to the insoluble fraction, confirming that it favors the misfolded/aggregated state. In addition, the LC3II/LC3I ratio was not only significantly greater in Mut-PrP expressing cells (Figure 5(c), black bars) but also was the highest within the insoluble fraction (Figure 5(c), grey bars), suggesting that LC3II within the autophagosome membrane may become incorporated with the aggregated PrP cargo it surrounds, similar to that recently reported with synuclein [34].

### 3.5. Ultrastructural Markers of Autophagy in Mut-PrP Expressing Cells

**Figure 6:**
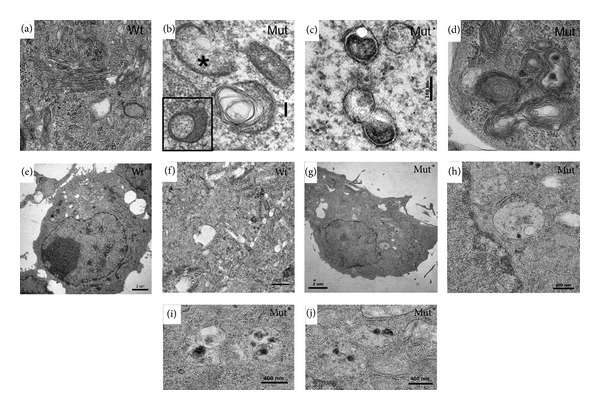
Ultrastructural markers of autophagy with Mut-PrP expression. Transmission electron micrographs of transiently transfected COS-7 cells ((a)–(d)) and stably transfected N2a cells ((e)–(j)). TEM of COS-7 cells expressing Wt (a) or Mut- ((b)–(d)) PrP for 24 h. Normal appearing ultrastructure present in cells expressing Wt-PrP (a), and abnormal membranes characteristic of isolation membranes ((b), asterisk), double membrane autophagosomes (c), and myelinoid (multilamellar) bodies (d) were evident in the majority of cell sections sampled from Mut-PrP expressing cells. (e)–(j) Mouse neuroblastoma N2a cells stably expressing Wt-PrP ((e)–(f)) or Mut-PrP ((g)–(j)). Mut-PrP expressing cells displayed an accumulation of multivesicular bodies and autophagy-related structures consistent with late stage autophagosomes and/or autolysosomes. These structures were largely absent in cells expressing Wt-PrP.

A hallmark of autophagy is the presence of ultrastructural markers, notably double membrane vesicles characteristic of autophagosomes. We carried out transmission electron microscopy (TEM) on cells transfected with Wt- or Mut-PrP to document the presence of these markers. We studied two cell lines using two methods of PrP expression. COS-7 cells were transiently transfected with Wt-PrP (Figure 6(a)) or Mut-PrP (Figures 6(b)–6(d)), and N2a cells were stably expressing Wt-PrP (Figure 6(e)) or Mut-PrP (Figures 6(f)–6(j)). Although this was not a quantitative study, when compared with cells transiently expressing Wt-PrP (Figure 6(a)), double bilayer membrane vesicles measuring 0.1 to 0.4 *μ*m in diameter, characteristic of autophagosomes (Figures 6(b) and 6(c)), isolation membranes (Figure 6(b), asterisk), and myelinoid (multilamellar) bodies (Figures 6(b) and 6(d)), known features of autophagy [31, 35], were readily apparent in cells expressing Mut-PrP (Figures 6(b)–6(d)). In N2a cells stably expressing Mut-PrP, the majority (70%) of surveyed cells (*n* > 25) displayed multivesicular bodies (MVBs) (Figure 6(h)) and autophagosomes/autolysosomes that contained cargo (Figures 6(h), 6(i), and 6(j)). Such structures were sparse or absent in cells stably expressing Wt-PrP (Figures 6(e) and 6(f)). These observations agree with our data in Figure 5, which supports an induction of the autophagy pathway in Mut-PrP expressing cells.

### 3.6. Mutant PrP Colocalizes with LC-3 Labeled Vesicles

**Figure 7:**
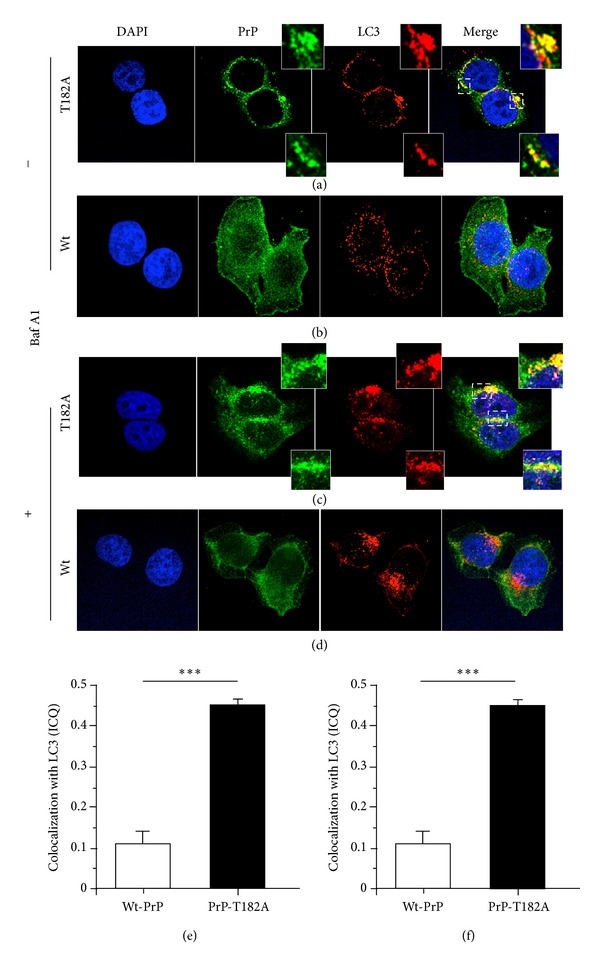
Mut-PrP colocalizes with autophagosomes. Confocal immunofluorescence of HeLa cells stably expressing GFP-tagged Mut- (a, c) or Wt- (b, d) PrP before (−) and after (+) incubation for 6 h with 50 nM Baf A1. Cells were fixed with 4% paraformaldehyde for 15 min, permeabilized with 0.1% Triton X-100 for 5 min, then stained with anti-LC3B antibody and DyLight 649-conjugated Affini-Pure goat anti-rabbit IgG, prior to inverted confocal fluorescence microscopy imaging using a Marianas Yokogawa type spinning disk (original magnification, x100). Li's method (NIH Image J plugin) was applied to determine the degree of colocalization of PrP::GFP (green) and LC3B (red). Graphs display the intensity correlation quotient (ICQ), which ranges from −0.5 (no correlation of signals) to 0.5 (complete correlation), between LC3B and Wt- or Mut-PrP in the absence (e) or presence (f) of BafA1. (***P < 0.0001). There were no differences in any values between those treated with BafA1 and control cells. Actual values are (e) BafA1(−) Wt-PrP = 0.110 ± 0.06, Mut-PrP = 0.450 ± 0.023, (f) BafA1(+) Wt-PrP = 0.117 ± 0.038, Mut-PrP = 0.447 ± 0.015,  *n* = 10 cells each.

We next assessed whether Mut-PrP could be detected within autophagosomes. To facilitate the study, we generated HeLa cell lines stably expressing Wt- or Mut-PrP::GFP, to which we applied simultaneous direct and indirect immunofluorescence to detect PrP::GFP and LC3, respectively (Figure 7). In Wt-PrP expressing cells, we found LC3 staining to be diffusely distributed throughout the cytosol, whereas in cells expressing Mut-PrP, its distribution was more polarized and overlapped closely with the intracellular distribution of Mut-PrP (Figures 7(a), 7(b), and 7(e)). Pretreatment of cells with Bafilomycin A1, to inhibit autophagosome fusion and degradation in lysosomes, did not alter the colocalization pattern of either Mut-PrP or Wt-PrP, despite significant enhancement of LC3 staining (Figures 7(c), 7(d), and 7(f)). Quantification of PrP colocalization with LC3, using Li's ICQ, was also similar in the presence or absence of Bafilomycin A1 and averaged 0.11 for Wt-PrP/LC3 and 0.45 for Mut-PrP/LC3 (Figures 7(e) and 7(f)), with a score of 0.5 representing maximum overlap of signal. This strongly suggests that Mut-PrP is specifically and closely associated with autophagosomal structures.

### 3.7. Autophagy Inhibition Impairs Delivery of Mut-PrP to LysoTracker Labeled Vesicles

**Figure 8:**
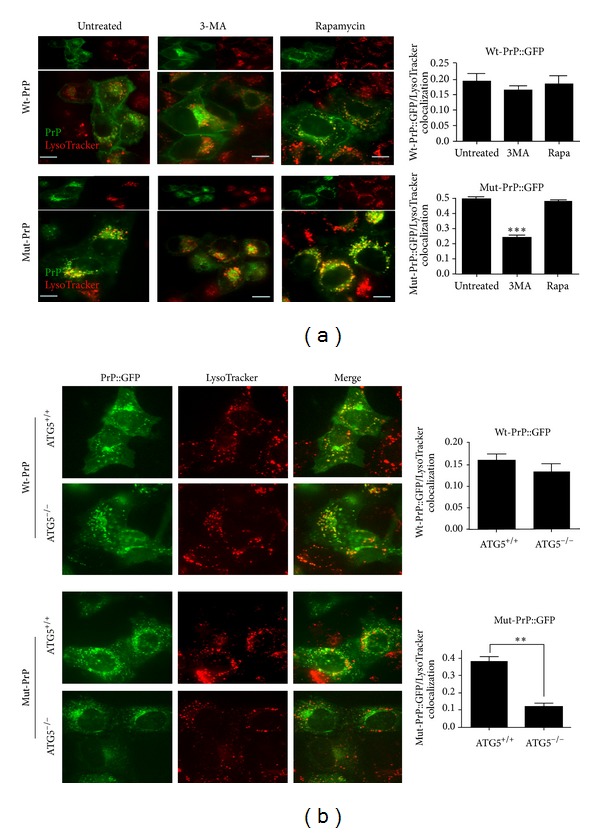
Autophagy participates in the delivery of Mut-PrP to LysoTracker labeled vesicles. (a) Three hours after transfection, HeLa cells expressing GFP-tagged Wt-PrP or Mut-PrP were treated for 16 h with 2 mM 3-MA, 2 *μ*M rapamycin, or fresh media alone (untreated), followed by incubation with 50 nM LysoTracker Red for 30 min prior to live cell confocal fluorescence microscopy. Individual channels are displayed above merged images. Yellow indicates colocalization. Scale bars = 10 *μ*m. Graphic display of Li's correlation coefficient between Mut-PrP and LysoTracker for each treatment is shown to the right (***P < 0.001, student's paired *t*-test, difference from untreated, *n* = 5 each). (b) Normal (ATG5^+/+^) and autophagy-deficient (ATG5^−/−^) mouse embryonic fibroblasts (MEFs) transiently transfected with GFP-tagged Wt-PrP or Mut-PrP were allowed to express for 16 h prior to confocal microscopy, performed 30 min following the addition of 50 nM LysoTracker Red. Single 0.5 *μ*m slice images are shown. Each channel is displayed to the left of the merged image. Graphic display of Li's correlation coefficient between PrP and LysoTracker Red in WT MEFs compared with ATG5^−/−^ MEFs is to the right (**P < 0.005, student's *t*-test, *n* = 5 each).

Our findings thus far suggested that Mut-PrP might induce autophagy as a mechanism for its delivery to lysosomes for degradation. To confirm the dependence of this pathway on autophagy, we determined if induction and/or inhibition of autophagy could modify the delivery of PrP to acidic vesicles labeled by LysoTracker Red. The relative colocalization of PrP::GFP with LysoTracker was, therefore, assessed following 16 h incubation with 2 mM 3-methyladenine (3-MA), a commonly used inhibitor of autophagy [36], or 2 *μ*M rapamycin, to induce autophagy [37]. For live cell imaging, we transiently transfected HeLa cells to avoid the confounding effect of the long half-life of Mut-PrP. Neither the distribution of Wt-PrP nor its colocalization with LysoTracker was significantly altered by either treatment although a small, but nonsignificant, reduction in colocalization following 3-MA was noted (ICQ = 0.19 ± 0.11 (CTL), 0.16 ± 0.06 (3-MA), 0.18 ± 0.11 (rapamycin), *P* = 0.653 for CTL versus 3-MA treated) (Figure 8(a), top row). In contrast, 3-MA significantly reduced Mut-PrP colocalization with LysoTracker, compared with untreated cells [ICQ = 0.48 ± 0.022 (CTL), 0.23 ± 0.048 (3-MA)] (Figure 8(a), bottom row). Interestingly, rapamycin did not enhance the already prominent colocalization of Mut-PrP with LysoTracker (Figure 8(a) bottom row and graph).

As an alternate method to assess the functional role of autophagy in the trafficking of Mut-PrP to LysoTracker labeled vesicles, we employed an autophagy-deficient mouse embryonic fibroblast (MEF) cell line that lacks Atg5 (i.e., ATG5^−/−^ MEFs), a key protein in the initiation of Atg5/Atg7-dependent macroautophagy [38] (Figure 8(b)). As in non-MEFs, Wt-PrP::GFP colocalized at a relatively low level with LysoTracker in wild type (WT) MEFs (ICQ 0.16 ± 0.06). Interestingly, in agreement with the 3-MA data in HeLa cells, a slight but nonsignificant reduction in the colocalization of Wt-PrP with LysoTracker was observed in ATG5^−/−^ MEFs (ICQ 0.13 ± 0.07, *P* = 0.782, *n* = 15, student's *t*-test), again suggesting that a small fraction of Wt-PrP might follow this route. As in the HeLa cells, Mut-PrP expressed in WT MEFs colocalized with LysoTracker to a greater extent than Wt-PrP (ICQ 0.38 ± 0.1), whereas, in ATG5^−/−^ MEFs, the reduction in colocalization was greater than that measured in 3-MA treated HeLa cells (ICQ 0.12 ± 0.06, *P* = 0.005). Interestingly, a small but detectable level of Mut-PrP did continue to colocalize with LysoTracker in ATG5^−/−^ MEFs, supporting a recent finding that these cells may have an Atg5-independent autophagy pathway [39] or that a secondary mechanism for delivery to lysosomes exists.

### 3.8. Autophagy Is Selective for Misfolded PrP

**Figure 9:**
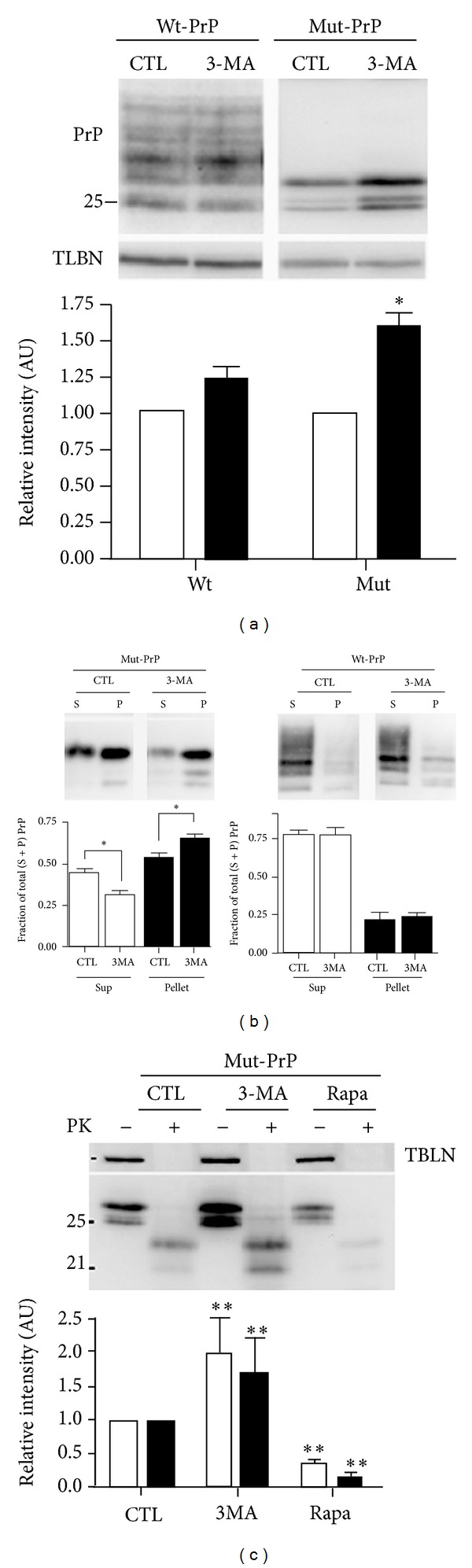
Autophagy is selective for insoluble/aggregated PrP. (a) Representative Western blot of total lysates from COS-7 cells transiently expressing Wt- or Mut-PrP (3F4) after 16 h of incubation with 5 mM 3-MA. The corresponding graph displays the relative densitometry of PrP signal before (open bars) and after (filled bars) 3-MA treatment (*P < 0.05, student's paired *t*-test, *n* = 3). Tubulin (TBLN) is a loading control. (b) Representative Western blots of supernatant (S) and pellet (P) fractions of cells transiently expressing Mut or Wt PrP after 16 h of vehicle (CTL) or 10 mM 3-MA, and the corresponding graph from 3 experiments each. The insoluble (pellet) and soluble (sup) fractions of Mut-PrP are presented as the densitometric signal of each fraction over the sum of the two fractions (sup + pellet) ± SEM (*P < 0.05, 2-way ANOVA, Bonferroni posttest). (c) Cells transiently expressing Mut-PrP were treated for 6 h with 10 mM 3-MA or 20 *μ*M rapamycin, harvested in lysis buffer, and digested with 5 *μ*g/mL Proteinase-K (PK) for 15 min at 37°C. Densitometry quantified as the intensity of each fraction (i.e., total PrP (open bar) or PK-resistant PrP (filled bar)), relative to their respective untreated normalized control fractions (**P < 0.005 ANOVA, Bonferroni post test, difference from CTL, *n* = 3 experiments).

To confirm that autophagy plays a functional role in the delivery of intracellular Mut-PrP to lysosomes, we assessed whether inhibiting autophagy alters the steady state levels of Mut-PrP. To test this, we again used COS-7 cells because they lack endogenous PrP and transient transfection was used to affect treatments early in the expression of PrP. Thus, 8 h from the transfection of Mut- or Wt-PrP, cells were treated with 5 mM 3-MA overnight (16 h). This resulted in significantly increased levels of Mut-PrP, but not Wt-PrP (*P* < 0.05) (Figure 9(a)). Because we noted earlier that LC3II partitioned with the insoluble fraction of Mut-PrP (see Figure 5), we questioned whether inhibiting autophagy with 3-MA primarily affects the insoluble fraction of Mut-PrP on the way to the lysosome. To assess this, cells were treated as described above and then detergent lysed and centrifuged at 16,000 ×g, to separate soluble and insoluble PrP. Comparing the ratio of soluble to insoluble PrP fractions revealed a significant shift of Mut-PrP, but not Wt-PrP, from the soluble to insoluble fraction (Figure 9(b)), suggesting autophagy primarily functions to eliminate the misfolded/aggregated fraction of PrP.

Based on these results, we questioned whether autophagy directly affects the *de novo* generation of pathogenic Proteinase-K resistant Mut-PrP^Sc^. The T182A mutant of PrP is well known to develop partial resistance to PK when expressed in cultured cells [19, 23]. Since 3-MA inhibits the clearance of misfolded/insoluble PrP, the fraction predicted to be the precursor to PrP^Sc^, we considered that autophagy acts to reduce the spontaneous generation of PK-resistant Mut-PrP. Thus, COS-7 cells were transfected as described above and treated for 6 h with either 10 mM 3-MA or 20 *μ*M rapamycin, followed by the digestion of lysates with 5 *μ*g/mL PK for 15 min at 37°C. In the presence of 3-MA, an increase in the level of Mut-PrP was accompanied by a proportional increase in PK-resistant PrP, whereas rapamycin reduced PK-resistant PrP to negligible levels (Figure 9(c)).

## 4. Discussion

Here we show for the first time the autophagy features in the intracellular trafficking and the degradation of newly synthesized misfolded/aggregated mutant PrP. This was revealed using an extensively studied PrP mutant linked to familial CJD and known to display aberrant trafficking and intracellular accumulation. We found that the T182A Mut-PrP was not localizable to the plasma membrane, it rapidly formed intracellular aggregates, and it colocalized with LysoTracker/LAMP-1 positive vesicles in a Golgi-independent manner, sooner, and to a greater extent, than Wt-PrP. Mut-PrP expression was also associated with an elevation in several markers of the autophagy-lysosomal pathway, the presence of ultrastructural markers of autophagy, and it extensively colocalized with the autophagosome-specific marker, LC3B. As a functional correlate, we found the delivery of Mut-PrP to lysosomes was profoundly impaired in autophagy-deficient ATG5^−/−^ MEFs and in normal cells treated with the autophagy inhibitor 3-MA. The latter also selectively impaired the degradation of the insoluble fraction of Mut-PrP, leading to an increase in the amount of PK-resistant Mut-PrP.

Autophagy has been shown to play a role in several long-lived proteins associated with neurodegenerative disease, including huntingtin [9], synuclein [10], and beta-amyloid (A*β*) [8]. In general, autophagy appears to function as an alternative means to clear cytoplasmic protein aggregates too large to fit within the pore of the proteasome, which supports the idea that impaired autophagy might contribute to the development of neurodegenerative disease [40]. Based on our current findings with a Mut-PrP prone to aggregation and retention in the ER [19], autophagy also appears to function as an early quality control mechanism that links the ER to lysosomes. Such a route has been suggested with other aggregation-prone proteins, including *α*1-antitrypsin Z mutant [41, 42], vasopressin [43], and dysferlin [44]. Although we did not address the potential role of the proteasome, it was previously reported that the T182A mutant of PrP is not significantly affected by proteasome inhibition [23], which is likely a consequence of its propensity to rapidly misfold and aggregate within the ER, thereby limiting its ability to unfold and be retrotranslocated to the cytosol. Thus, another outlet, such as autophagy, is required for its elimination.

Ashok and Hegde [45] reported that some disease-linked mutated PrPs traffic to lysosomes from the trans-Golgi, although the mechanism of transport has not been defined. Despite misfolding in the ER, the C-terminal domain mutations of PrP they studied were found to traffic to the Golgi prior to their degradation in Bafilomycin A1 sensitive compartments (most likely lysosomes). Although similar to our proposed pathway, the PrP mutants they studied were not recognized by ER quality control and, importantly, they reached the trans-Golgi, as defined by their Endo H resistance, a key distinction from the Endo H sensitive T182A mutant that does not traffic beyond the mid-Golgi stack. Our finding that Golgi disruption by Brefeldin A did not prevent delivery of Mut-PrP to acidic compartments further supports a Golgi-independent process in its delivery to lysosomes.

This report is not the first to link autophagy to PrP or prion disease. Oh et al. [46] found that cultured hippocampal neurons lacking PrP (*Prnp* ^−/−^) displayed increased levels of LC3II and autophagy-related EM ultrastructures relative to *Prnp* ^+/+^ neurons, and the reintroduction of PrP^C^ into the cells reverted them to the wild type phenotype. They suggested that the reduction in endogenous PrP^C^ that occurs in prion disease, as a result of its conversion to PrP^Sc^, could activate autophagy. While our studies do not rule out autophagy induction during later stages of prion disease, when PrP^C^ levels could be significantly reduced, we show that autophagy might function as an early quality control mechanism to eliminate misfolded/aggregated PrP that accumulates in the ER. As such, this process may be especially important in genetic prion diseases, to limit the accumulation of mutated PrP that has a high propensity to spontaneously misfold in the ER.

Although the mechanism for induction of autophagy has not been determined, we hypothesize that ER stress induced by accumulation of PrP aggregates within the ER is the trigger. This is supported not only by the results with Mut-PrP, but also by the finding that Brefeldin A, which limits Wt-PrP exit from the ER and increases its insoluble fraction, promoted trafficking of PrP to lysosomes. Interestingly, Brefeldin A is a powerful inducer of ER stress and the unfolded protein response (UPR), suggesting a link between UPR and autophagic delivery of PrP to lysosomes. Prior work supports the induction of ER stress and the unfolded protein response (UPR) in cell culture models of prion disease and in brain samples derived from patients with CJD [47, 48], in addition to mice infected with prions [49]. Importantly, recent evidence also links the induction of autophagy with ER stress-induced UPR [50, 51]. Bernales et al. [52] found that DTT-mediated induction of the UPR in yeast caused ER swelling and the accumulation of autophagic vesicles delimited by ribosome-studded ER membranes. We hypothesize that a similar process in mammalian cells might lead to the sequestration of accumulating PrP aggregates within the ER. This is further supported by a recent work suggesting that the ER membrane is the origin of at least some types of mammalian autophagic isolation membranes [29].

In addition to quality control of misfolded secretory proteins, the autophagic pathway we outline here could have a secondary function specific to prion disease, which is to limit the *de novo* generation of PrP^Sc^. We found that inhibiting autophagy increased the absolute level of insoluble PrP, suggesting that normally or partially folded PrP^C^ may be recruited to form misfolded/aggregated PrP if the pool of misfolded PrP is not efficiently removed by autophagy. Because misfolded/aggregated PrP is thought to contribute directly to the generation of PrP^Sc^, autophagy could then act to limit the *de novo* generation of PrP^Sc^. This is underscored by the finding that the inhibition of autophagy in cells expressing Mut-PrP resulted in a proportionate increase in the overall level of PK-resistant PrP, while induction of autophagy reduced it. This compares well with the work of others, who found that induction of autophagy using several agents, including lithium, trehalose, imatinib (i.e., Gleevec), and rapamycin, reduced the levels of PrP^Sc^ in chronically prion-infected cell lines, and in some cases, *in vivo * [11, 12, 53, 54]. Thus, as with poly-Q expanded mutant huntingtin protein [55], autophagy might play a protective role in prion disease. In fact, we confirmed that the protective effect of rapamycin in Huntington disease mouse models [56] also translated to an improved survival and reduced plaque deposition in our Tg (PrP-A116V) mouse model of genetic prion disease [13].

While these data suggest that the manipulation of the autophagy pathway be an important therapeutic target for many neurodegenerative proteinopathies, it is important to be cautious when designing autophagy therapeutics targeting such diseases. Recent reports indicate that autophagy induction could also result in neurotoxic effects, especially in disorders in which autophagy flux (full progression through the autophagy pathway), and not autophagy initiation, is impaired. Such flux impairments have been reported for Huntington disease and Alzheimer's disease [57, 58]. It is noteworthy that prions and misfolded mutant PrP, including this T182A PrP mutant, have been reported to be released into the media of cultured cells, and, as such, have the potential to infect neighboring cells. In fact, PrP-T182A was found to accumulate intracellularly, possibly within lysosomes, in heterologous cells exposed to secreted material [23]. Our results in general, and especially the finding that Brefeldin A treatment enhanced colocalization of PrP-T182A within acidic vesicles labeled by LysoTracker, suggest two important principles: first, the delivery of the mutant PrP to this compartment is primarily via an intracellular pathway, since Brefeldin A should effectively block the secretory pathway, and second, the secretion of this protein is likely a result of MVB release via exosomes, as reported for *bona fide* prions in prion-infected cultured cells [59, 60], or alternatively, directly from lysosomes via lysosomal exocytosis [61]. It is, therefore, cautioned that autophagy induction in prion disease, with little or no concomitant increases in lysosomal clearance of PrP, could conceivably result in enhanced prion spread. Although our prior results in Tg(PrP-A116V) mice and the current results presented here in cultured cells, proved beneficial, caution is still advised. More research into genetic prion disease, the role played by autophagy plays in such disorders, as well as the similarities and differences to acquired prion diseases, is needed.

## 5. Conclusion

Our studies provide for an early quality control mechanism that employs autophagy to eliminate newly synthesized misfolded aggregated PrP from the ER that might otherwise contribute to the formation of pathogenic PrP^Sc^. This model provides new opportunities to understand the nature of prion generation and propagation, in addition to other proteins that may utilize this pathway. Whether manipulating autophagy can alter the course of human prion disease will be of a great interest in the design and development of possible treatments, especially in the case of genetic prion disease.

## Supplementary Material

Video Supplement S1. Time-lapse fluorescence microscopy of Mut-PrP. Maximum intensity projection of time-lapsed confocal z-stack sequence for a COS-7 cell observed every 10 min from the first detection of Mut-PrP::GFP (~ 5 h) for 2 h and displayed at 3 fps. Cells were located using the EM-CCD, as the expression level was poorly visible to the eye. Note that the earliest detection of Mut-PrP is in the form of puncta, suggesting aggregates of PrP. This is supported by the finding that these puncta increase in intensity and size over the observation period. Also note the perinuclear accumulation of puncta. This movie corresponds to Figure 3A.
Video Supplement S2. Time-lapse fluorescence microscopy of Mut-PrP with LysoTracker Red. Images are as described in Video Supplement 1. Acidic vesicles are labeled with LysoTracker Red. Images were collected every 10 min over a 140 min observation period, beginning at T=7 h (420 min) and displayed at 3 fps. Colocalized pixels are rendered solid yellow to aid visualization. This corresponds to Figure 3B.
Video Supplement S3. Time-lapse fluorescence microscopy of Wt-PrP with LysoTracker Red. At T=3 h, cells were imaged every 10 min for 950 min. Images are maximum intensity projections using only the central optical slices of the cell (to visualize internal details), created by trimming the z-stack data using ImageJ software. Image display rate is 3 fps. This corresponds to Figure 3C.
